# Cortico-subcortical converging organization at rest

**DOI:** 10.1038/s41598-025-18023-9

**Published:** 2025-09-01

**Authors:** Dheemant Jallepalli, Shilpa Dang

**Affiliations:** 1 Network & Cognitive Neuroscience Lab, Centre for Brain Science & Applications, School of Artificial Intelligence & Data Science, Indian Institute of Technology Jodhpur, Jodhpur, India; 2 Department of Biomedical Engineering, Carnegie Mellon University, Pittsburgh, PA USA

**Keywords:** Cortico-subcortical circuits, Integration, Network hubs, Resting state fMRI, Resting state network, Segregation, Neural circuits, Time series

## Abstract

Local segregation and global integration are the fundamnetal organizational principles of human brain. It is unknown how subcortex configures itself with respect to the segregation and integration dynamics at rest. Using resting state functional MRI data of 92 healthy adult participants, we revealed three non-overlapping segregated communities in subcortex, confining anatomically to thalamus, basal ganglia, and subcortical limbic structures, termed as subcortical networks. Further using network science, we analysed the topology of subcortex and found about 80% of subcortical regions acting as hubs, connecting with other cortical as well as subcortical communities. Next, using statistical modelling, we determined the role of subcortex (both at region-level and network-level) in cortical information integration and found multiple, widespread cortical regions (networks) converging onto individual subcortical regions (networks) (a many-to-one mapping). Individual subcortical networks showed varied extent of convergence, broadly from primary and association networks in cortex. We found functional diversity of cortex to be the major driving factor behind cortical convergence within subcortex and that the absence of subcortical regions significantly impacted the information transmission efficiency within the cortico-subcortical converging organization. Overall, our results provide insights into the subcortical organization at rest and underscore the subcortical contributions in shaping the large-scale brain dynamics.

**Supplementary Information:**

The online version contains supplementary material available at 10.1038/s41598-025-18023-9.

## Introduction

**Fig. 1:**
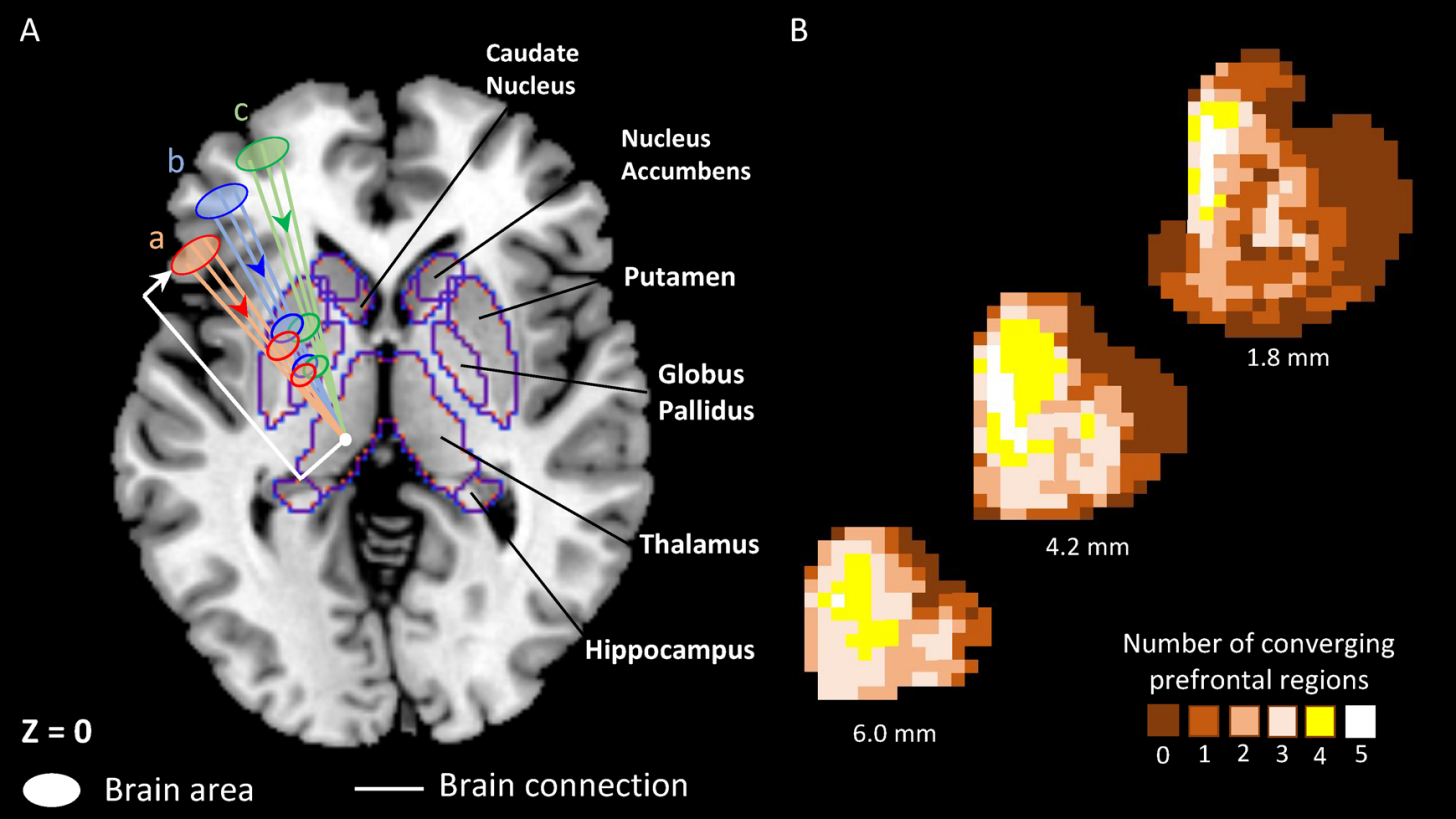
Evidence from past. (**A)** Generalized schematic diagram based on the parallel organization of functionally segregated circuits^7^. Cortical connections from areas (a, b, c) partially converge in the dorsal striatum (a structure of basal ganglia consisting of putamen and caudate nucleus). The striatal regions send further converging connections to incrementally smaller areas such as globus pallidus, which in turn project to a specific region of the thalamus. The outputs from these basal ganglia structures loop back to the cortex via thalamus in parallel but segregated basal ganglia – thalamocortical channels, specifically named as motor circuit, oculomotor circuit, lateral orbitofrontal circuit, dorsolateral prefrontal circuit, and anterior cingulate circuit^7^. Arrows indicate direction of the brain connection. (**B)** Heatmaps of cortico-striatal convergence areas in rhesus macaques, based on data from an invasive tract-tracing experiment^18^. These heatmaps represent voxel-wise converging projections in the striatum from 0 to 5 distinct prefrontal cortical (PFC) regions (i.e., ventromedial PFC, orbitofrontal cortex, dorsal PFC, ventrolateral PFC, dorsal anterior cingulate cortex). Shown here are striatal slices at 6.0 mm, 4.2 mm, and 1.8 mm, anterior to anterior commissure (recreated representative slices with permission).

The human subcortex lies deep within the brain and comprises ~ 8% of brain mass but contains only 0.8% of neurons in human brain^1^. Despite its size, subcortex consists of diverse neural structures, including thalamus, basal ganglia, hippocampus and amygdala. These structures have been assumed, in past, to simply subserve cortex^2^. Contrary to this perspective, subcortical structures perform specific functions^3–6^ through subcortical and cortico-subcortical circuits^7–9^. These circuits are parallelly organized, functionally segregated, and linked with goal-directed and habitual behaviours^7,10,11^ (Fig. 1A). However, higher-order cognitive functions such as goal-directed behaviours cannot occur in isolation, instead they rely on integration of information between various specialized neural communities^9,12–15^. Previous research indicates that both local segregation and global integration exist as fundamental organizational principles in human cortex^14–16^. Further, past studies have shown convergence from functionally diverse cortical sites within the subcortex^17–19^. Moreover, for both functional segregation and integration, the subcortex serves as an important neuroanatomical substrate due to its key anatomical features, including progressive compression of pathways over incrementally smaller structures^20^ and proximity of many smaller nuclei^1^ (Fig. 1A). We believe that these features are notably beneficial for information integration, as it allows for synaptic contact and thus, communication between proximal subcortical regions of distinct functional systems^7,10,21^.

Despite above findings, it is unknown how subcortex configures itself at rest with respect to the local segregation and global integration dynamics^14,22^. Unlike prevalent research on cortical resting state networks (RSNs)^23,24^ the available knowledge on the resting state organization of subcortex is limited^25,26^ and fragmented with respect to cortical networks^27–31^. Cerliani et al. (2015), using resting state functional magnetic resonance imaging (rs-fMRI) have investigated functional connectivity between subcortical and cortical RSNs in healthy vs. autism spectrum disorder conditions. These networks were identified using independent component analysis and revealed one subcortical RSN, including basal ganglia and thalamus, whereas amygdala as part of a cortical network including temporal pole and pons. However, there was no mention of hippocampus in the study. Using rs-fMRI and a top-down hierarchical clustering approach^32^, Tian et al. (2020) have revealed a multi-scale atlas of subcortex, starting from entire subcortex as one node and hierarchically revealing 54 subcortical parcels or regions at the finest scale. Please note that the contributions of our study contrast with the studies employing top-down hierarchical parcellation schemes yielding functional parcels of subcortex^25,33^. Specifically, we used the 54 functional parcels of subcortex^25^ as the starting point and a *bottom-up* hierarchical clustering approach^32^ to obtain the subcortical networks at rest.

Despite existing studies, we require further investigation into the resting state organization of subcortex. Using rs-fMRI data, functional connectivity, and bottom-up hierarchical clustering approach^32^we first aimed to identify the subcortical networks at rest. We define these networks as non-overlapping sets of subcortical regions exhibiting similar subcortico-subcortical functional connectivity, based on a past approach employed for the cerebral cortex^23,28^. Next, to quantify the role of subcortex with respect to the local segregation and global integration principles, we used network science^34^ and compared the topological characteristics of subcortical networks against the cortical networks. Lastly, we aimed to understand how such segregated organization in subcortex would participate in convergence at global scale. Please note that by “convergence” here, we mean integration of information from multiple regions at a particular site^30^ i.e., a many-to-one mapping where multiple cortical regions have connections with a particular subcortical region. We do not indicate any sort of directionality or causal flow of information here. There is ample evidence from past anatomical tract-tracing and single-cell recording studies on how convergence from diverse cortical sites occurs within the subcortex^7,18^ shown in Fig. 1. Additionally, several functional neuroimaging studies have focused on understanding the cortico-subcortical correlations during resting state^28–31^ providing evidence that spontaneous neural activity in subcortex can be fractionated into several independent components correlating with activity in cortical networks. Strikingly, these neuroimaging studies have adopted a cortico-centric approach and focussed on delineating the network-level components within subcortex (for instance, identifying (one-to-one) mapping between specific cortical network and subcortical region) rather than revealing the organization of region-wise cortical mapping to subcortical regions.

Specifically, we aimed to determine the cortico-subcortical converging organization at rest by addressing the following questions: (1) what is the extent of cortical convergence within subcortex – by computing the exact number of distinct cortical regions converging onto subcortical regions, (2) what causes these cortical regions to converge – by examining how convergence between the cortical regions was related to the distance and type of nodes (functionally diverse or similar) in the cortex, and (3) what makes subcortex important for convergence – by simulating a targeted attack on subcortical nodes and assessing its potential impact on information transmission efficiency within the converging organization. Overall, our study aims to advance the existing knowledge of subcortical organization at rest by revealing its segregated architecture and most importantly, the region-by-region cortico-subcortical converging organization at rest. Elucidating the cortico-subcortical convergence at rest is crucial for two overarching goals of the field – one, to understand how efficient brain function is based on successful communication between subcortex and cortex and two, how alterations in this communication may lead to various diseased states^11,35,36^.

## Results

### Resting state networks of subcortex

**Fig. 2:**
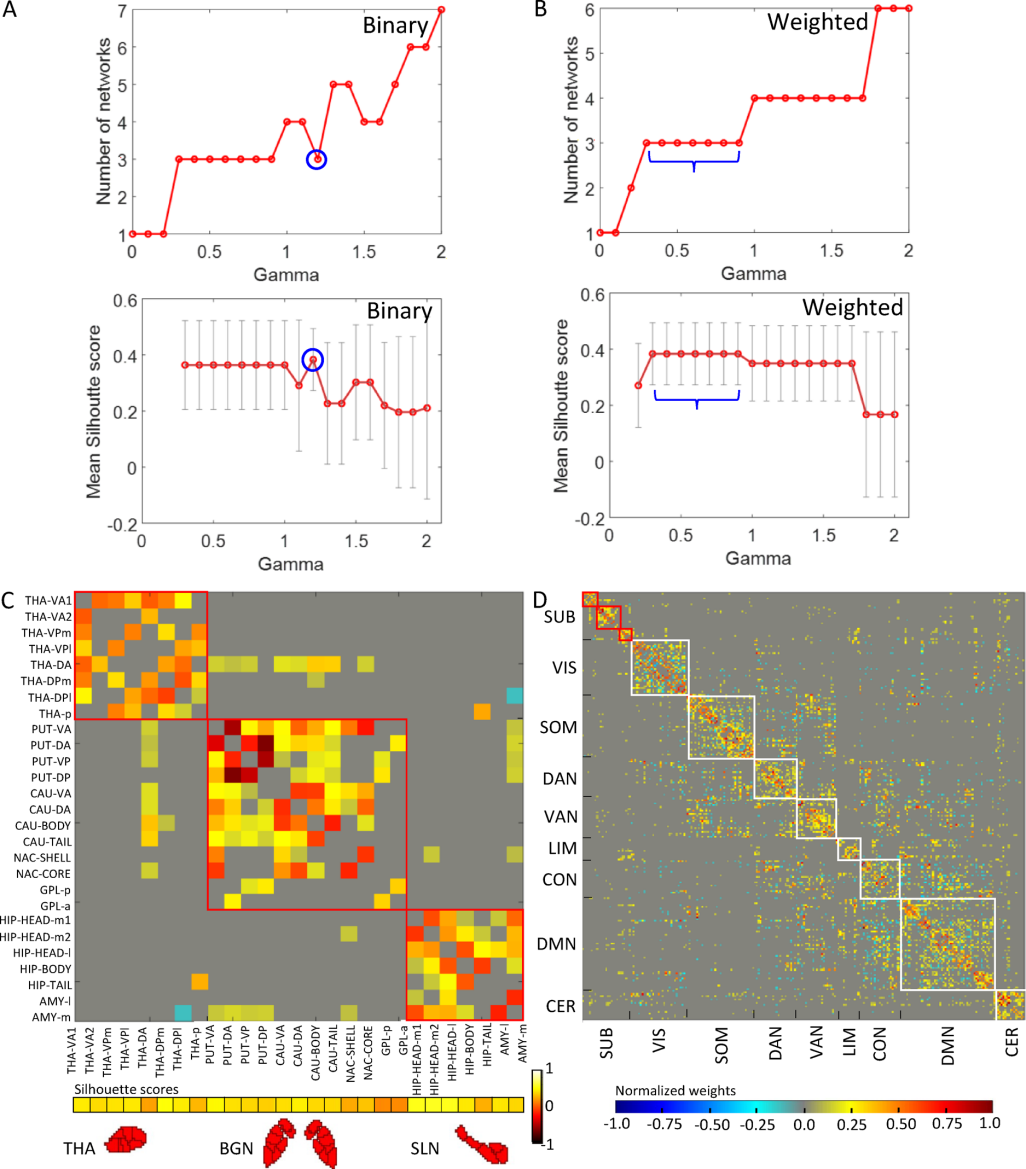
Subcortical and cortical networks at rest. **(A-B)** Louvain clustering algorithm results employed on group-level binary and weighted functional connectivity matrices, showing number of networks (communitites) identified in subcortex and corresponding network assignment confidence score (mean Silhouette score) for $\:\gamma\:\:$values ranging from 0 to 2 in steps of 0.1. The most optimal clustering solution was obtained corresponding to the highest network assignment confidence score value of 0.38 (± 0.11), both for $\:\gamma\:=1.2$ in case of binary FC matrix and for $\:0.3\le\:\gamma\:\le\:0.9$ in case of weighted FC matrix (marked in panels **A-B**). (**C)** Group-level ROI-by-ROI weighted functional connectivity matrix based on partial correlation method, sorted by three communities identified within the subcortex, i.e. the thalamic network (THA, comprising of all thalamus ROIs), the basal ganglia network (BGN, comprising of all putamen, caudate nucleus, nucleus accumbens, and globus pallidus ROIs) and the subcortical limbic network (SLN, comprising of all hippocampus and amygdala ROIs), see Supplementary Fig. S5 (ROIs: V – ventral, D – dorsal, A or a – anterior, P or p – posterior, l – lateral, m – medial). The block-like pattern along the diagonal indicates a modular organization in subcortex where connections within a community are stronger than connections between the communities. The horizontal bar (at the bottom) represents the node-wise Silhouette scores (ranging from $\:-1.0\:to\:1.0$) of 27 subcortical ROIs. **D** Similar matrix shown at whole brain level, sorted according to the three newly identified subcortical communities (SUB) and eight existing cortical communities from literature^23,24^ – visual network (VIS), somatomotor network (SOM), dorsal attention network (DAN), ventral attention network (VAN), limbic network (LIM), control network (CON), and default mode network (DMN). The horizontal jet colormap (at the bottom) represents the strength of normalized weights (ranging from − 1.0 to 1.0) for both the panels A and B, significant at corr. *p* < 0.05.

We determined the segregated communities in subcortex at rest using group-level functional connectivity (FC) of (*N* =) 27 subcortical nodes or regions-of-interest (ROIs) (see Methods, Regions-of-interest). The ROIs were formed by combining bilateral parcels (see Supplementary Fig. S1 and Methods, Whole brain functional parcels). The FC was computed using *partial correlation* between ROIs by using a multiple linear regression model^37^. Partial correlation captures unique dependence between any two nodes while controlling for the effects of all other nodes in consideration (i.e. subcortical as well as cortical nodes) (see Methods, Functional connectivity). Next, we employed Louvain clustering algorithm^38^ that identified communities by maximizing modularity, i.e. stronger connections within the community in comparison to connections between the communities (see Methods, Louvain clustering algorithm). We refer to these segregated communities as subcortical networks (see Fig. 2), which are non-overlapping sets of subcortical ROIs exhibiting similar subcortico-subcortical functional connectivity, as has been done in past for cortex^23,28^. Figure 2A-B show results of clustering algorithm employed separately on both the group-level binary and weighted FC matrices of subcortex, for $\:\gamma\:$ values ranging from 0 to 2. The panels 2A and 2B show the number of networks (communities) identified in subcortex and their corresponding network assignment confidence scores (mean Silhouette score) at individual $\:\gamma\:$ values, respectively. The most optimal clustering solution was obtained corresponding to the highest network assignment confidence score value of 0.38 (± 0.11), both for $\:\gamma\:=1.2$ in case of binary FC matrix and for $\:0.3\le\:\gamma\:\le\:0.9$ in case of weighted FC matrix (marked in Fig. 2A-B). Interestingly, both the group-level binary and weighted FC matrices yielded same final partitioning scheme as the best solution.

The clustering analysis revealed 3 functionally segregated communities in the subcortex (Fig. 2). Notably, these communities were consistent with the well-known anatomical structures of the subcortex, i.e. the thalamic network (THA) comprising of all thalamus (THA) nodes, the basal ganglia network (BGN) comprising of all putamen (PUT), caudate nucleus (CAU), nucleus accumbens (NAC), and globus pallidus (GPL) nodes, and the subcortical limbic network (SLN) comprising of all hippocampus (HIP) and amygdala (AMY) nodes. This is along the lines of recent research indicating that the anatomical architecture of the brain shapes and constrains its functional activity^39,40^. Figure 2C-D show the group-level ROI-by-ROI weighted FC matrix for the subcortex and the whole brain, sorted by community assignment. The block-like patterns in the matrix indicate that the subcortical RS organization exhibited modularity, i.e. stronger partial correlation within-community than between-community (statistics reported in next section along with other metrics). This was similar to the existing RS organization found in cortex^23,24^. Moreover, for comparison we also computed the group-level pair-wise full correlations between subcortical nodes and found no clear block-like pattern for the identified communities (more details included in Supplementary Information & Fig. S2). This indicated that the partial correlation reliably detected the true connectivity between nodes^37^ and thus, in turn the true modular organization.

Further, we assessed if the observed partial correlation between any two subcortical nodes is statistically significant and not a spurious effect due to their internal temporal structure, i.e. autocorrelation effects^41^. We employed a nonparametric permutation testing approach using surrogate data^42^. For this, we generated 100 surrogate versions of the original time series that preserved their autocorrelation structure by using phase-randomized surrogates but destroyed any genuine cross-series dependency (more details included in Supplementary Information & Fig. S3). We found that the observed partial correlation results reflected true cross-dependence between the nodes and were significantly greater than pure autocorrelation effects ($\:p<0.01,\:t\left[{df}_{1}+{df}_{2}\right]=88.45,\:{df}_{1}={N}^{2}-1,\:\:{df}_{2}={100N}^{2}-1)$).

For preliminary validation, silhouette score (SS) was used. SS indicates the confidence score of a node’s affiliation with the assigned community (see Methods, Silhouette score). We found that the scores for subcortical nodes were all positive and significantly greater than zero (mean SS (± SD) = 0.38 (± 0.11), *t*[26] = 18.04, *p* = 10^−16^, horizontal bar at bottom of Fig. 2A), indicating an appropriate clustering solution (see Supplementary Fig. S4 and Table S1 for further validation results of clustering solution).

### Network validation

Further, statistics-based network analysis^43,44^ was used to validate whether the characteristics of the segregated organization in subcortex were significantly different than chance. For this, a comparison of real network was done with 1000 random “null” networks (see Methods, Statistical network validation). The random networks were generated by rewiring connections between nodes while maintaining the same degree distribution as our “representative” subcortical network organization at rest (obtained above). This is a commonly used approach for network validation^15,43,45,46^. Characteristic graph theory metrics were computed for the real and random networks both at node-level and network-level, including measures of segregation (within-module degree (WMD), modularity index $\:Q$) and those of integration (participation coefficient (PC), characteristic path length (CPL)) (see Methods, Statistical network validation for definition on each metric). We included integration metrics to check whether our community assignment results were affected by nodes with substantial between-module connections that contradict the hypothesis of segregated RSNs at rest.

**Fig. 3:**
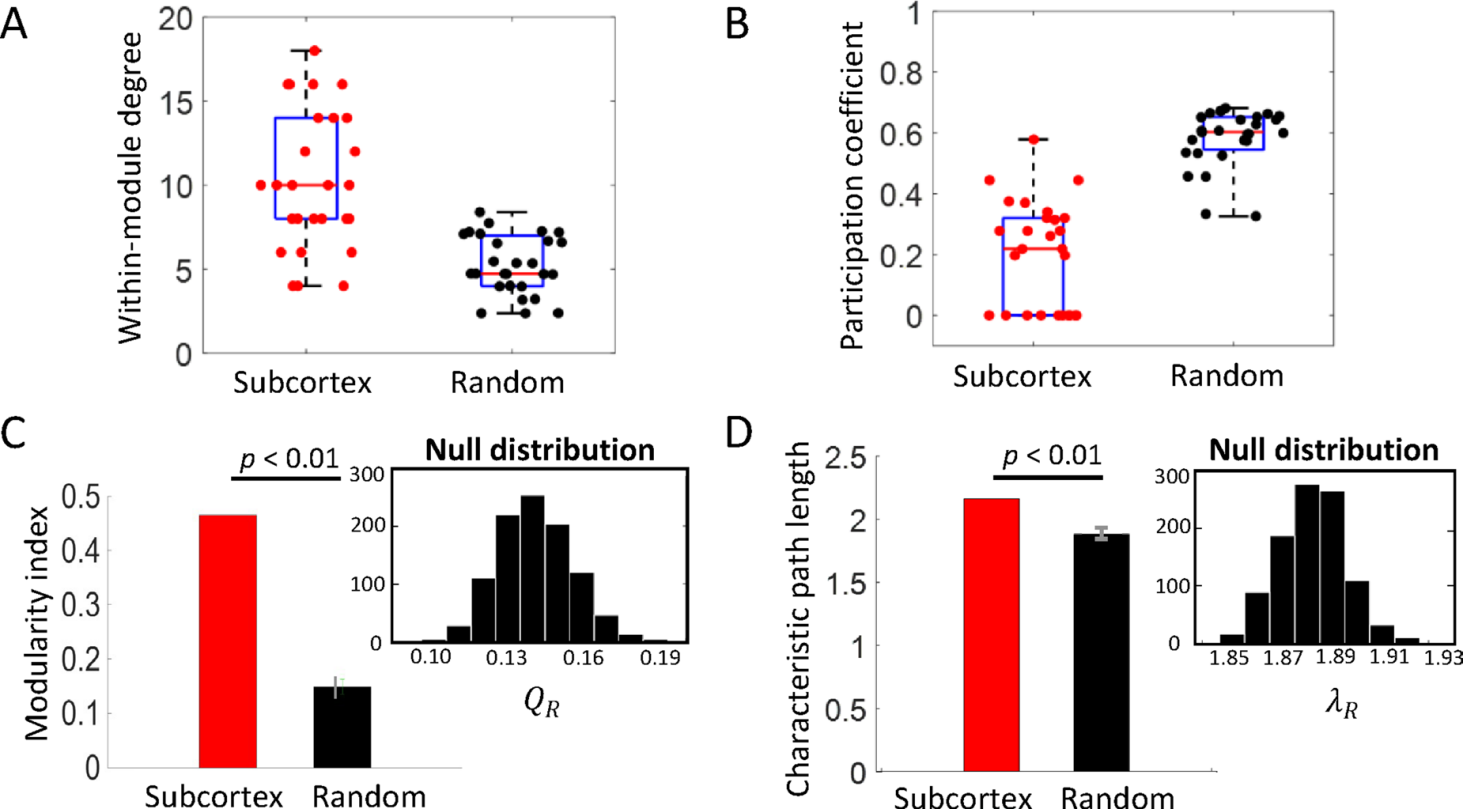
Characteristic graph metric values across subcortex and random networks. (**A-B)** Node-level metrics. Higher within-module degree (WMD) and lower participation coefficient (PC) of 27 subcortical ROIs (red dots) than their counterpart nodes in random network (black dots), illustrating significant difference in the topology of subcortex in comparison to random (R) network (mean $\:{wmd}_{i}$ (± SD) = 10.52 (± 3.90), mean $\:{wmd}_{iR}$ (± SD) = 5.64(± 2.43), mean $\:{P}_{i}$ (± SD) = 0.20 (± 0.18), mean $\:{P}_{iR}$ (± SD) = 0.58 (± 0.13); all corr. *p*-values < 0.01, $\:i\:=\:1\:to\:27$, see Supplementary Table S1 for node-wise statistics). Please note that the PCs have been calculated with respect to subcortical communities only. Each red dot represents metric value of one node in the subcortex, and each black dot represents the mean metric value of the “null” distribution obtained from a population of 1000 random networks (see Methods for statistical test used). On each box, the central mark (solid red line) is the median, the edges of the box are the 25th and 75th percentiles, the whiskers extend to the maximum and minimum datapoints. (**C**-**D)** Network level metrics. Higher modularity index $\:Q$ and characteristic path length $\:\lambda\:$ of subcortex network (red bars) than random network (black bars), illustrating stronger modular organization in subcortex than expected in random networks ($\:Q$ = 0.47, mean $\:{Q}_{R}$ (± SD) = 0.15 (±0.01); *t*[999] = 752.85, corr. *p* < 0.01; $\:\lambda\:$ = 2.18, mean $\:{\lambda\:}_{R}$ (± SD) = 1.88 (±0.01); *t*[999] = 733.74, corr. *p* < 0.01). Black bar in each main panel represents mean metric value of the “null” distribution obtained from a population of 1000 random networks (see inset panel). Error bars (in grey) indicate standard deviation of metric values across random networks.

We found that the topological characteristics of the subcortex were significantly different than expected in a “null” distribution obtained from a population of 1000 random topologies^43,45^. Figure 3 shows a comparison of characteristic graph metrics of segregation and integration across subcortex and random networks. For node-level metrics WMD and PC, we observed a significant difference between the subcortical and random nodes, i.e. the subcortical nodes were well-connected to nodes of their own community in comparison to nodes of other communities (see Methods for statistical test used). This was indicated by higher WMD values and lower PC values of subcortical nodes than their counterparts in random networks (R) (see Fig. 3A, B, mean $\:{wmd}_{i}$ (± SD) = 10.52 (± 3.90), mean $\:{wmd}_{iR}$ (± SD) = 5.64(± 2.43), mean $\:{P}_{i}$ (± SD) = 0.20 (± 0.18), mean $\:{P}_{iR}$ (± SD) = 0.58 (± 0.13); all corr. *p*-values < 0.01, $\:i\:=\:1\:to\:27$, see Supplementary Table S1 for node-wise statistics). For network-level metrics modularity $\:Q$ and characteristic path length $\:\lambda\:$, again we found that the subcortex exhibited both higher modularity and higher characteristic path length in comparison to the random networks, ($\:Q$ = 0.47, mean $\:{Q}_{R}$ (± SD) = 0.15 (±0.01); *t*[999] = 752.85, corr. *p* < 0.01; $\:\lambda\:$ = 2.18, mean $\:{\lambda\:}_{R}$ (± SD) = 1.88 (±0.01); *t*[999] = 733.74, corr. *p* < 0.01). These validation results indicated the reliability of the identified modular organization in subcortex.

## Subcortical hubs

**Fig. 4:**
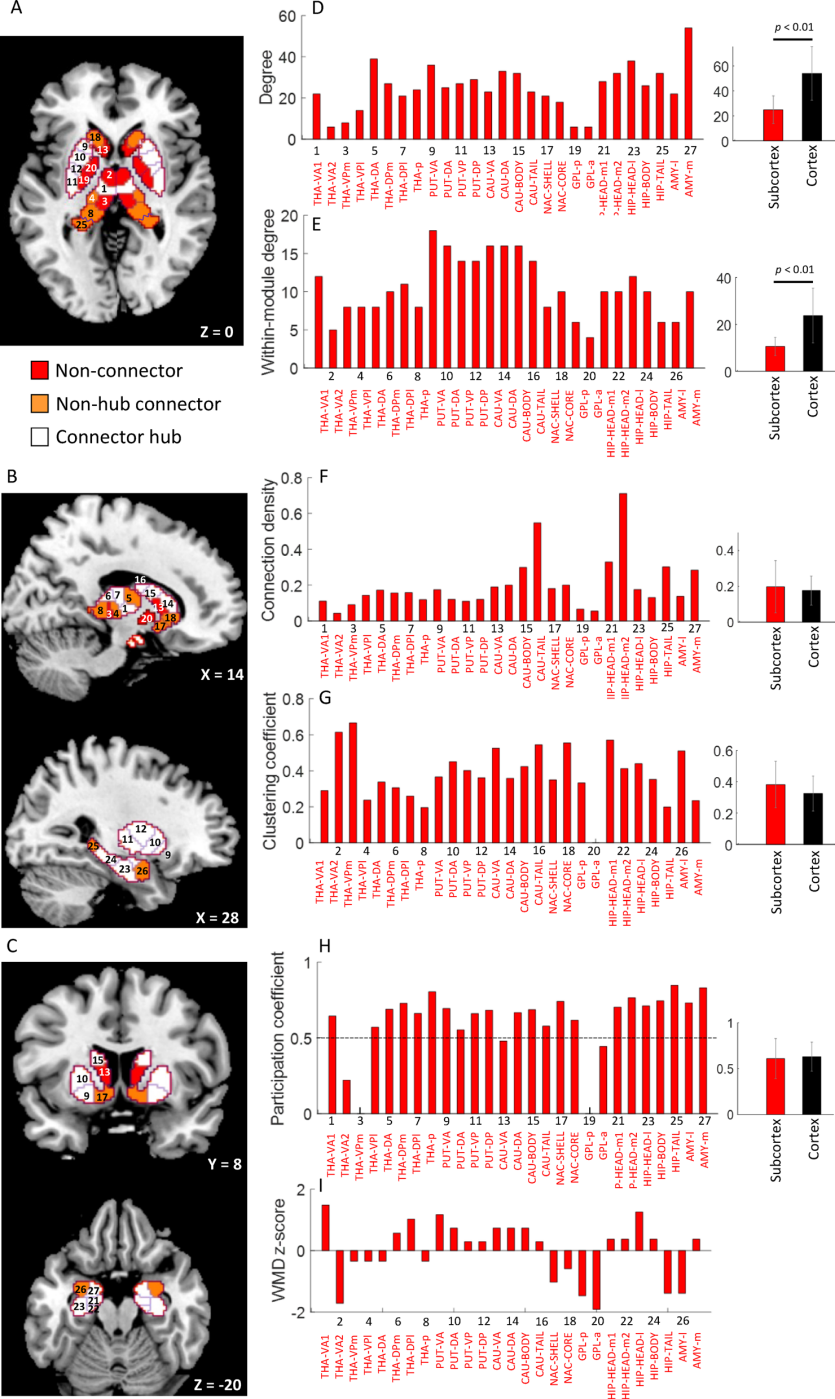
Topology of subcortex and graph metrics. **(A-C)** Distinct types of nodes in the subcortex crucial for balancing the local segregation and global integration dynamics – the non-connectors (in red) including 4 non-hubs (THA-VA2, THA-VPm, GPL-p, GPL-a) and 1 provincial hub (CAU-VA), the latter type are important for specialised functional computations, 7 non-hub connectors for global information integration only (in orange; THA-VPl, THA-DA, THA-p, NAC-shell, NAC-core, HIP-tail, AMY-l), and 15 connector hubs for both functional segregation and integration (in white; THA-VA1, THA-DPm, THA-DPl, PUT-VA, PUT-DA, PUT-VP, PUT-DP, CAU-DA, CAU-body, CAU-tail, HIP-head-m1, HIP-head-m2, HIP-head-l, HIP-body, AMY-m). Numbers $\:1-27\:$ are shown node-wise for reference. (**D-I)** Node-wise values of (within-module) degree, connection density, clustering coefficient, participation coefficient, and within-module degree z-score of each of the 27 nodes in the subcortex. Also shown in right-most panels is the comparison across the topology of subcortex (27 nodes) and cortex (217 nodes). The bars in rightmost panels of D-I represent mean values across respective nodes. Error bars (in grey) indicate standard deviation of metric values across nodes. Significant difference across graph metrics of subcortex and cortex was determined using a two-sample t-test with equal means and equal but unknown variances at *p* < 0.01 (see Supplementary Table S2 for individual graph metrics’ statistics). Please note that the PCs have been calculated with respect to whole brain communities.

We examined the topology of subcortex to understand its role in the whole brain organization at rest. For this, we identified four distinct types of nodes, including connector hubs, non-hub connectors, provincial hubs, and non-hubs, based on a fundamental work^47^ (see Methods, Topology of subcortex for definition on each node type). Hubs are brain regions with high degree that play a key role in information processing for functional segregation and integration^15,45^. We found that about 81.48% of nodes in subcortex (27 nodes) were connectors (15 connector hubs and 7 non-hub connectors) and remaining 18.52% were non-connector type (1 provincial hub and 4 non-hubs) (see Fig. 4A-C). Further, we found a similar distribution in cortex (217 nodes), as 82.03%:17.97%. Moreover, we found that the topological characteristics, including connection density (degree per voxel), clustering, and participation coefficients were comparable across subcortex and cortex (see Fig. 4D-I and Supplementary Table S2; for more details and definitions on characteristic graph metrics refer Methods, Statistical network validation section). Thus, our results here show that despite of its smaller nuclei structures, subcortex supports both segregation and integration equivalent to the cortex.

## Cortico-subcortical converging organization at rest

### Extent of convergence within subcortex

**Fig. 5:**
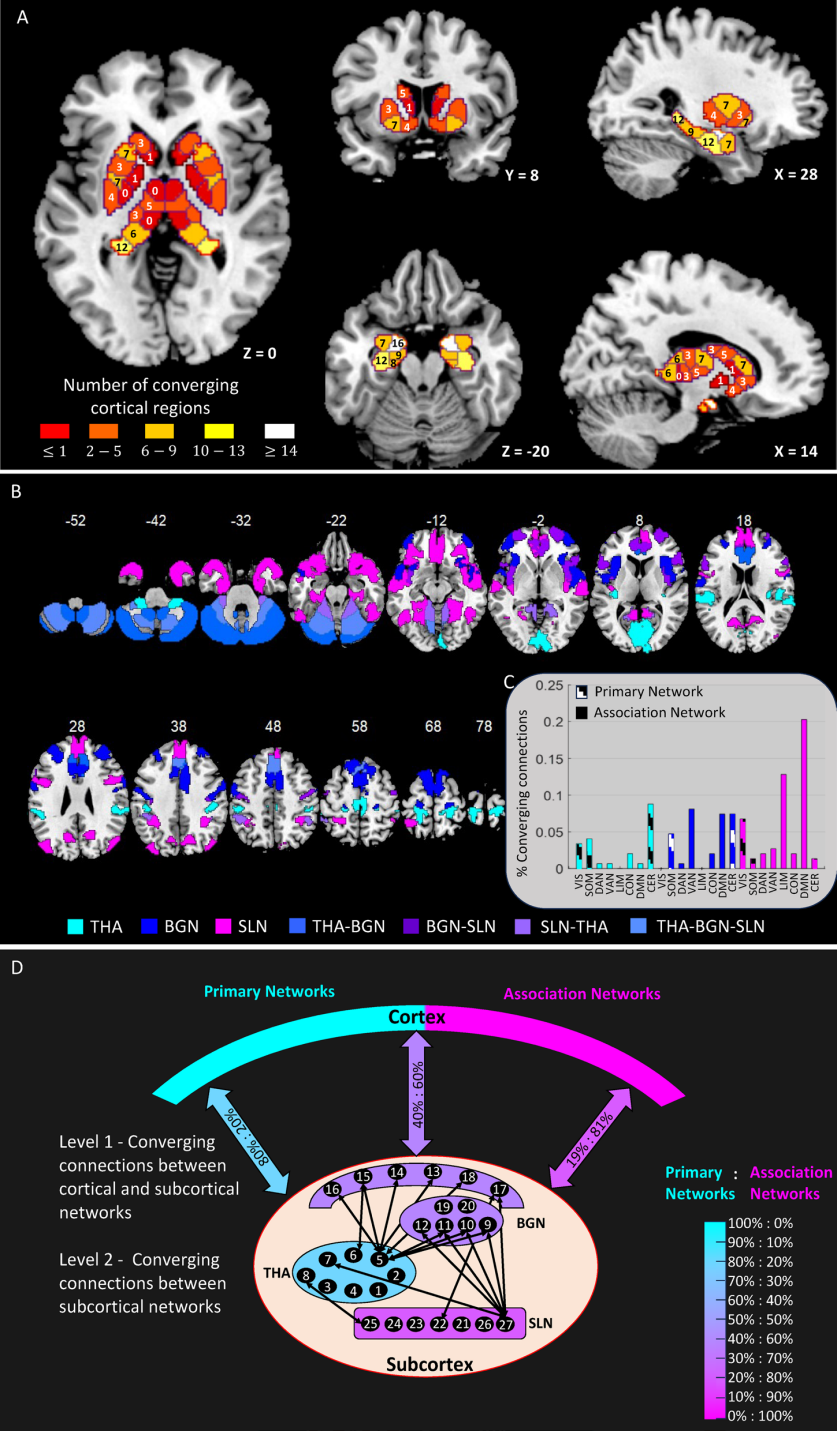
Cortico-subcortical converging organization at rest. **(A)** Areas of convergence in the human subcortex. Each sub-panel illustrates the number of distinct cortical regions that converge onto each of the 27 subcortical regions-of-interest through bidirectional connections. The colour of each subcortical ROI represents the degree of convergence, while the overlaid number indicates the exact count of converging inputs. (**B)** Cortical regions converging onto subcortical ROIs are shown at a significance threshold of *p* < 0.01, corrected for multiple comparisons using the Bonferroni method. For clearer visualization, these are displayed separately for the thalamic network (THA, cyan), basal ganglia network (BGN, blue), and subcortical limbic network (SLN, magenta). Certain cortical regions converge onto ROIs belonging to multiple subcortical networks and are distinctly color-coded using shades of blue and purple, as indicated at the bottom of panel B. Axial slices shown at different z-coordinates (See Fig. S5 for sagittal and coronal views). (**C)** Percentage of total converging (bidirectional) connections between cortical and subcortical resting-state networks. The proportions of converging inputs to subcortical networks—THA, BGN, and SLN—are represented by distinct coloured bars (cyan, blue, and magenta, respectively). Inputs from primary networks, including early sensory and late motor areas as well as the cerebellum, are shown as patterned bars, while those from association networks are shown as solid bars. (**D)** Schematic representation of large-scale cortico-subcortical convergence at rest. Level 1 illustrates bidirectional converging connections (coloured arrows) from various cortical networks—grouped into primary (cyan) and association (magenta) networks—to subcortical networks. Specifically, the thalamic network (THA) receives 80% of its inputs from primary and 20% from association networks; the basal ganglia network (BGN) receives 40% and 60%, respectively; and the subcortical limbic network (SLN) receives 19% from primary and 81% from association networks. The colormap (right) reflects the percentage ratio of convergence from primary vs. association networks within each subcortical network (ranging from 0%:100–100%:0%). Level 2 depicts additional convergence (black bidirectional arrows) between subcortical networks (refer to Fig. 2; within-network connections are excluded here). The numbers 1–27 correspond to the 27 subcortical ROIs. Bidirectional arrows denote connectivity.

We quantified the extent of cortical convergence within subcortex by computing the absolute number of cortical ROIs converging onto each of the 27 subcortical ROIs (see Methods, Convergence & distance metrics). This analysis not only revealed information about how many cortical regions converged but also specifically where that integration of information occurred in subcortex and among which of the cortical ROIs (see Fig. 5; Table 1, and for details on individual converging cortical regions refer Supplementary Table S3). The absolute number of cortical (bidirectional) connections onto a subcortical ROI ranged from $\:0-16$, where non-connectors received at most from one cortical site and connectors received from multiple cortical sites (see Fig. 5A).

Next, we quantified the extent of convergence at network-level to reveal how connections from specific cortical networks mapped to specific subcortical networks. In the resting state organization of cerebral cortex, it has been shown that the primary sensory and motor cortices, i.e. visual and somatomotor networks, are separated from the association networks^23^. To quantify the extent of large-scale network convergence within subcortex, we categorised the eight cortical networks (including cerebellum) into primary and association networks, where primary networks included visual network, somatomotor network, and cerebellum^23^. The association networks included the dorsal attention, ventral attention, limbic, control, and default mode networks^23^. Specifically, we computed the percentage ratio of the extent of convergence from primary networks to the extent of convergence from association networks within a particular subcortical network (see Fig. 5B-C). The thalamic network received its major convergence (about 80% of its connections) from primary networks, i.e. VIS, SOM, and CER, and remaining 20% from association networks DAN, VAN, CON, and DMN (see Fig. 5D and see Table 1 for details). Further, the basal ganglia network had major convergence (about 60% of its connections) from DAN, VAN, CON, and DMN and remaining 40% from motor cortex and cerebellum. Interestingly, no connections were received from the sensory and limbic cortices. Finally, the subcortical limbic network, including hippocampus and amygdala received majority (about 81%) of its connections from association networks (mainly LIM and DMN) and remaining 19% from primary networks (mainly VIS). Interestingly, the hippocampus (HIP-Tail) was the only subcortical region that received at least one connection from all cortical networks.

Together, our results revealed not only the segregated organization of subcortex at rest but also how this organization participated in convergence at global scale (Fig. 5). Our results indicated that the fundamental organizational principles of local segregation and global integration exist at the level of subcortex as well. Further, the convergence occurred from multiple, widespread, and diverse cortical networks (nodes), and systematically mapped onto individual subcortical networks (Fig. 5D). On one hand, the thalamic network received majority of convergence from primary networks whereas on the other hand, subcortical limbic network received majority of convergence from association networks. The basal ganglia network lied intermediate to the other two subcortical networks in terms of ratio of converging connections received from primary and association networks. The subcortex further demonstrated convergence between its networks THA, BGN, and SLN, as shown in Fig. 5D.

**Table 1:** Converging cortical regions within subcortex

ROI index	Subcortical ROI	Number of converging connections	Converging cortical ROIs*	Centroids (x, y,z) (in mm) of bilateral parcels forming a cortical ROI	RSN	T(91)**
1	THA-VA1	5	V1 Area 4 (M1) Area p24 (PreACC) CER 6 CER 9	(−5,−88,2), (8,−92,−2) (−4,−25,56), (4,−25,58) (−5,34,20), (7,35,25) (−22,−61,−24), (26,−60,−25) (−10,−51,−48), (11,−51,−48)	VIS SOM DMN CER CER	4.51 4.21 4.51 5.66 7.20
2	THA-VA2	0	-	-	-	-
3	THA-VPm	0	-	-	-	-
4	THA-VPl	3	OP1 (S2) Area 3b (S1) Area 2 (S1)	(−48,−24,18), (49,−21,18) (−18,−31,69), (22,−29,68) (−46,−29,44), (45,−28,43)	SOM SOM DAN	5.75 4.99 4.96
5	THA-DA	7	Area 8BM (dmPFC) CER Crus 1 CER Crus 2 CER 6 CER 10 Vermis 4,5 Vermis 6	(−4,28,47), (5,28,48) (−35,−68,−31), (39,−69,−31) (−27,−75,−40), (33,−71,−42) (−22,−61,−24), (26,−60,−25) (27,−36,−43), (21,−36,−44) (2,−54,−8) (2,−69,−17)	CON CER CER CER CER CER CER	4.59 4.22 8.93 5.69 5.44 4.30 4.51
6	THA-DPm	6	Area 3b (S1) Area 8BM (dmPFC) CER Crus 1 CER 8 CER 9 CER 10	(−18,−31,69), (22,−29,68) (−4,28,47), (5,28,48) (−35,−68,−31), (39,−69,−31) (−25,−56,−50), (26,−58,−51) (−10,−51,−48), (11,−51,−48) (27,−36,−43), (21,−36,−44)	SOM CON CER CER CER CER	4.25 5.32 4.11 4.13 4.81 5.09
7	THA-DPl	3	V1 V1 A1	(−7,−74,10), (9,−73,8) (−5,−88,2), (8,−92,−2) (−36,−24,10), (35,−22,14)	VIS VIS SOM	4.61 4.81 4.48
8	THA-p	6	V2 POS1 (VC) Area 2 (S1) PF (IPL) PF (IPL) CER 9	(−13,−43,−5), (18,−45,−3) (−14,−49,4), (14,−47,4) (−19,−40,72), (22,−35,70) (−55,−32,22), (58,−31,24) (−45,−41,47), (47,−44,47) (−10,−51,−48), (11,−51,−48)	VIS VIS SOM VAN CON CER	8.03 7.99 4.75 5.03 5.35 7.17
9	PUT-VA	7	Area 6r (SMA) AVI FOP Area a24pr (MCC) Area a47r (vlPFC) Area 9 m (dmPFC) Area p24 (PreACC)	(−49,6,26), (49,8,25) (−33,25,−1), (37,23,5) (−36,4,11), (38,7,10) (−6,22,31), (7,19,35) (−42,49,−7), (42,51,−6) (−6,45,6), (8,42,4) (−5,34,20), (7,35,25)	DAN VAN VAN VAN CON DMN DMN	4.16 4.22 4.40 5.82 −4.08 4.91 4.95
10	PUT-DA	3	Area 4 (M1) FOP SCEF (PMC)	(−59,−2,24), (61,6,29) (−50,1,4), (49,5,4) (−5,9,48), (8,2,43)	SOM VAN VAN	4.90 4.27 4.84
11	PUT-VP	4	Area 55b (PMC) Area 4 (M1) Area 4 (M1) CER 6	(−51,−7,43), (52,−6,37) (−41,−14,48), (44,−11,48) (−19,−24,66), (21,−24,66) (−22,−61,−24), (26,−60,−25)	SOM SOM SOM CER	4.57 9.62 5.61 7.24
12	PUT-DP	7	Area 4 (M1) Area 4 (M1) Area 4 (M1) PoI FOP Area 6ma (SMA) CER 6	(−59,−2,24), (61,6,29) (−41,−14,48), (44,−11,48) (−4,−26,69), (5,−22,72) (−39,2,−5), (39,−2,6) (−50,1,4), (49,5,4) (−8,−3,71), (7,−2,67) (−22,−61,−24), (26,−60,−25)	SOM SOM SOM VAN VAN VAN CER	4.79 5.42 4.10 4.88 4.19 5.90 7.97
13	CAU-VA	1	FOP	(−33,19,8), (37,23,5)	VAN	4.95
14	CAU-DA	7	SCEF (SMA) Area a10p (dlPFC) Area p24 (PreACC) SFL (SMA) SFL (SMA) CER Crus 1 CER Crus 2	(−5,9,48), (8,2,43) (−28,58,−1), (27,59,3) (−5,34,20), (7,35,25) (−13,24,61), (12,20,63) (−7,10,64), (6,11,57) (−35,−68,−31), (39,−69,−31) (−27,−75,−40), (33,−71,−42)	VAN DMN DMN DMN DMN CER CER	4.45 5.38 5.65 4.11 6.18 4.27 5.28
15	CAU-Body	5	AVI Area 8BM (dmPFC) CER 6 CER Crus 1 CER Crus 2	(−33,25,−1), (37,23,5) (−4,28,47), (5,28,48) (−22,−61,−24), (26,−60,−25) (−35,−68,−31), (39,−69,−31) (−27,−75,−40), (33,−71,−42)	VAN CON CER CER CER	4.48 5.06 4.85 5.92 5.77
16	CAU-Tail	3	STGa CER 6 CER Crus 1	(−53,6,−12), (53,3,−6) (−22,−61,−24), (26,−60,−25) (−35,−68,−31), (39,−69,−31)	DMN CER CER	−4.34 4.23 6.01
17	NAC-Shell	4	AVI Area 9 m (dmPFC) Area p24 (PreACC) CER 9	(−33,16,−8), (34,21,−8) (−6,45,6), (8,42,4) (−5,34,20), (7,35,25) (−10,−51,−48), (11,−51,−48)	CON DMN DMN CER	4.81 10.01 4.91 4.73
18	NAC-Core	3	Area 9-46d (dlPFC) Area 9 m (dmPFC) Area p24 (PreACC)	(−29,43,30), (32,45,28) (−6,45,6), (8,42,4) (−5,34,20), (7,35,25)	VAN DMN DMN	4.31 7.46 6.16
19	GPL-p	0	-	-	-	-
20	GPL-a	1	CER 8	(−25,−56,−50), (26,−58,−51)	CER	4.22
21	HIP-Head-m1	9	PHC POS1 (VC) PerC EntC STSda PGp (IPL) Area 10r (vmPFC) POS1 (pCunPCC) POS1 (pCunPCC)	(−30,−33,−18), (31,−31,−18) (−14,−49,4), (14,−47,4) (−26,−9,−33), (28,−1,−40) (−21,−21,−26), (22,−18,−27) (−57,−9,−14), (55,−4,−14) (−40,−79,30), (45,−75,31) (−6,35,−9), (5,40,−10) (−8,−52,9), (6,−52,23) (−13,−61,19), (13,−55,16)	VIS VIS LIM LIM DMN DMN DMN DMN DMN	4.59 7.72 6.49 9.64 4.91 4.55 6.06 5.03 7.86
22	HIP-Head-m2	8	POS1 (VC) TGd EntC STSda PGp (IPL) Area 10r (vmPFC) POS1 (pCunPCC) POS1 (pCunPCC)	(−14,−49,4), (14,−47,4) (−32,12,−30), (29,12,−31) (−21,−21,−26), (22,−18,−27) (−57,−9,−14), (55,−4,−14) (−40,−79,30), (45,−75,31) (−6,35,−9), (5,40,−10) (−8,−52,9), (6,−52,23) (−13,−61,19), (13,−55,16)	VIS LIM LIM DMN DMN DMN DMN DMN	6.07 4.99 15.92 4.27 4.52 5.06 4.24 6.74
23	HIP-Head-l	12	PHC PerC EntC TGd TGd STSda Area a47r (vlPFC) Area 10v (vmPFC) Area 10r (vmPFC) Area 9 m (dmPFC) POS1 (pCunPCC) POS1 (pCunPCC)	(−30,−33,−18), (31,−31,−18) (−26,−9,−33), (28,−1,−40) (−21,−21,−26), (22,−18,−27) (−43,13,−33), (49,9,−33) (−32,12,−30), (29,12,−31) (−57,−9,−14), (55,−4,−14) (−36,37,−13), (35,38,−14) (−5,55,−10), (5,40,−10) (−6,35,−9), (5,40,−10) (−6,45,6), (8,42,4) (−8,−52,9), (6,−52,23) (−13,−61,19), (13,−55,16)	VIS LIM LIM LIM LIM DMN DMN DMN DMN DMN DMN DMN	12.24 5.85 5.60 6.92 5.59 6.42 5.60 5.08 4.85 5.28 4.19 7.16
24	HIP-Body	9	PHC PHC TGd TGd Area 47 s (vlPFC) Area a10p (dlPFC) Area 9 m (dmPFC) Area p24 (PreACC) CER 4, 5	(−19,−37,−12), (18,−36,−13) (−30,−33,−18), (31,−31,−18) (−43,13,−33), (49,9,−33) (−32,12,−30), (29,12,−31) (−36,22,−15), (35,23,−17) (−28,58,−1), (27,59,3) (−6,45,6), (8,42,4) (−5,34,20), (7,35,25) (−14,−45,−19), (18,−45,−20)	VIS VIS LIM LIM DMN DMN DMN DMN CER	13.26 6.60 6.17 5.65 4.46 −4.91 4.30 4.15 5.73
25	HIP-Tail	12	PHC VMV1 V2 POS1 (VC) Area 2 (S1) AIP PoI PerC PF (IPL) AVI POS2 (pCunPCC) CER 8	(−30,−33,−18), (31,−31,−18) (−25,−54,−8), (26,−52,−9) (−13,−43,−5), (18,−45,−3) (−14,−49,4), (14,−47,4) (−30,−46,63), (31,−41,64) (−33,−46,41), (36,−45,45) (−39,2,−5), (39,−2,6) (−26,−9,−33), (28,−1,−40) (−45,−41,47), (47,−44,47) (−33,16,−8), (34,21,−8) (−10,−70,31), (13,−71,39) (−25,−56,−50), (26,−58,−51)	VIS VIS VIS VIS SOM DAN VAN LIM CON CON DMN CER	4.15 6.94 6.15 10.78 5.05 4.31 4.08 4.28 5.62 4.52 5.31 4.26
26	AMY-l	7	STGa PoI PerC TGd TGd TGd STSda	(−44,5,−16), (41,5,−16) (−40,−14,−2), (40,−11,−4) (−26,−9,−33), (28,−1,−40) (−25,6,−39), (28,−1,−40) (−43,13,−33), (49,9,−33) (−32,12,−30), (29,12,−31) (−57,−9,−14), (55,−4,−14)	VAN VAN LIM LIM LIM LIM DMN	4.75 4.44 4.23 4.56 8.40 11.14 4.38
27	AMY-m	16	Area 4 (M1) PH (TPOJ) Area 6r (SMA) STGa Area 13 L (OFC) PerC TGd TGd Area 8BM (dmPFC) STSda A5 (STG) Area 47 s (vlPFC) Area a47r (vlPFC) Area 10r (vmPFC) Area 44 (IFG) Area 9 m (dmPFC)	(−41,−14,48), (44,−11,48) (−49,−56,−16), (50,−49,−18) (−49,6,26), (49,8,25) (−44,5,−16), (41,5,−16) (−24,22,−20), (23,22,−20) (−26,−9,−33), (28,−1,−40) (−43,13,−33), (49,9,−33) (−32,12,−30), (29,12,−31) (−4,28,47), (5,28,48) (−57,−9,−14), (55,−4,−14) (−61,−13,−3), (62,−19,0) (−36,22,−15), (35,23,−17) (−36,37,−13), (35,38,−14) (−6,35,−9), (5,40,−10) (−53,19,11), (53,24,6) (−4,51,28), (6,58,29)	SOM DAN DAN VAN LIM LIM LIM LIM CON DMN DMN DMN DMN DMN DMN DMN	4.51 4.53 6.24 5.80 5.10 8.46 7.29 11.21 −4.89 6.67 4.89 5.22 5.24 5.17 −5.45 4.15

*ROI names from Glasser atlas^48^; see Supplementary Table S3 for individual functional roles and references; a – anterior, d – dorsal, l – lateral, m – medial, p – posterior, pr – prime, r – rostral, s – superior, v – ventral, A1 – Primary Auditory Cortex, A5 – Fifth Auditory Area, AIP – Anterior Intra Parietal, AVI – Anterior Ventral Insula, CER – Cerebellum, EntC – Entorhinal Cortex, FC – Frontal Cortex, FOP – Frontal Opercular, IFG – Inferior Frontal Gyrus, INS – Insula, IPL – Inferior Parietal Lobule, M1 – Primary Motor Cortex, MCC – Middle Cingulate Cortex, OFC – Orbitofrontal Cortex, OP – Parietal Operculum, PF – Parietal area F, PG – Parietal area G, pCunPCC – Precuneus-Posterior Cingulate Cortex, PerC – Perirhinal Cortex, PFC – Prefrontal Cortex, PHC – Parahippocampal Cortex, PMC – Premotor Cortex, PoI – Posterior Insula, POS – Parieto-Occipital Sulcus, PreACC – Pregenual Anterior Cingulate Cortex, S1 – Primary Somatosensory Cortex, S2 – Secondary Somatosensory Cortex, SCEF – Supplementary and Cingulate Eye Fields, SFL – Superior Frontal Language Area, SMA – Supplementary Motor Area, STG – Superior Temporal Gyrus, STS – Superior Temporal Sulcus, TPOJ – Temporo-Parieto-Occipital Junction, TG – Temporal Gyrus, V1 – Primary Visual Cortex, V2 – Second Visual Area, VC – Visual Cortex, VMV – Ventromedial Visual Area.

**Individual T-value T(91) reported for a cortical ROI having significant FC with a subcortical ROI computed at group-level (92 participants, random effects analysis (RFX) one-sample *t*-test at corr. *p <* 0.01, see Methods on Functional connectivity).

## Functional diversity driving cortical regions to converge

**Fig. 6:**
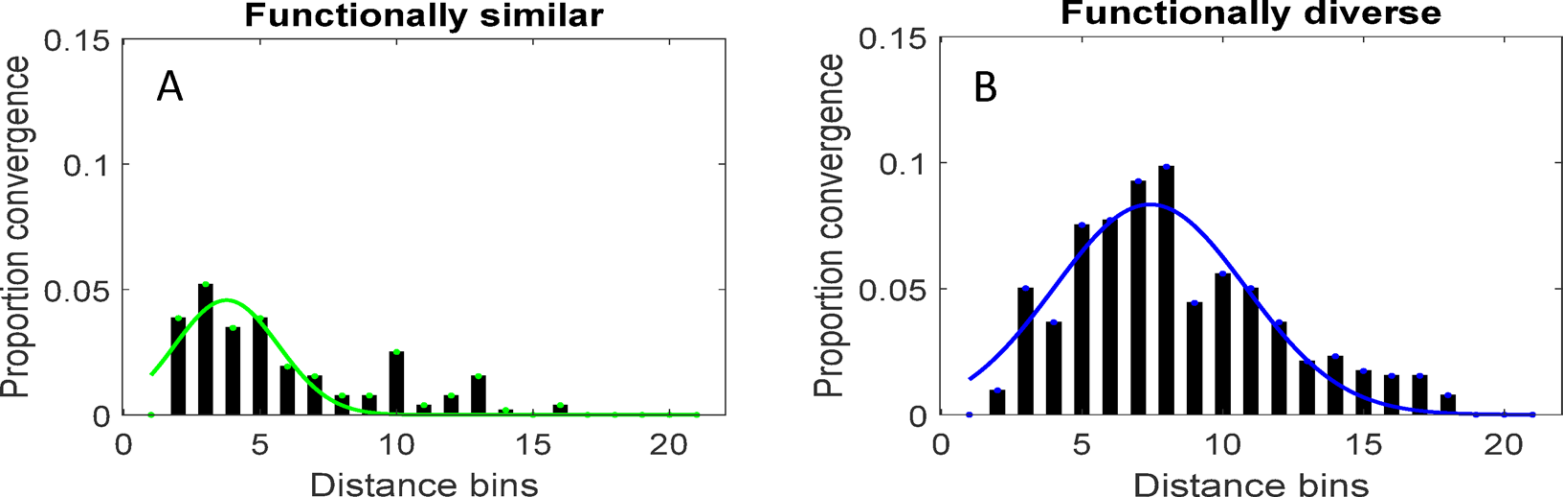
Normal distribution of distances between converging cortical regions. **(A**) Distribution representing proportion convergence for functionally similar cortical node pairs divided into bins of size 8 mm and approximated using normal function $\:\mathcal{N}({\mu\:}_{FS},{\sigma\:}_{FS}^{2})$ where, $\:{\mu\:}_{FS}=33.18\:mm,\:{\sigma\:}_{FS}=22.58\:mm$ (solid green curve). (**B)** Distribution same as in A for functionally diverse cortical node pairs and approximated using normal function $\:\mathcal{N}({\mu\:}_{FD},{\sigma\:}_{FD}^{2})$ where, $\:{\mu\:}_{FD}=60.10\:mm,\:{\sigma\:}_{FD}=28.19\:mm$ (solid blue curve). A cortical node pair is functionally similar, if both its nodes belong to a same resting state network and is functionally diverse, if its nodes belong to distinct resting state networks.

To determine how convergence was related to distance and node type, we obtained distributions of distances between the converging cortical node pairs of two types – functionally similar (FS) and functionally diverse (FD). A cortical node pair is FS, if both its nodes belong to a same RSN and is FD, otherwise, i.e. if its nodes belong to distinct RSNs. For this, we computed the Euclidean distance and the proportion convergence for a cortical node pair (see Methods, Convergence & distance metrics section for more details). Proportion convergence captures how often two nodes project simultaneously to the subcortical nodes with respect to the total convergence. We found that the distributions could be well approximated with normal functions (Fig. 6), $\:\mathcal{N}({\mu\:}_{FS},{\sigma\:}_{FS}^{2})$ where, $\:{\mu\:}_{FS}=33.18\:mm,\:{\sigma\:}_{FS}=22.58\:mm$ for FS node pairs and $\:\mathcal{N}({\mu\:}_{FD},{\sigma\:}_{FD}^{2})\:$ where, $\:{\mu\:}_{FD}=60.10\:mm,\:{\sigma\:}_{FD}=28.19\:mm$ for FD node pairs. The proportion convergence strengths of FD nodes were significantly greater than those of FS nodes (paired *t-*test of bin-wise strengths, *t*[20] = 3.52, *p* = 0.0022). Majorly, we found that the cortical regions from functionally diverse communities participated in the converging organization at rest.

### Subcortical nodes under targeted attack

**Fig. 7:**
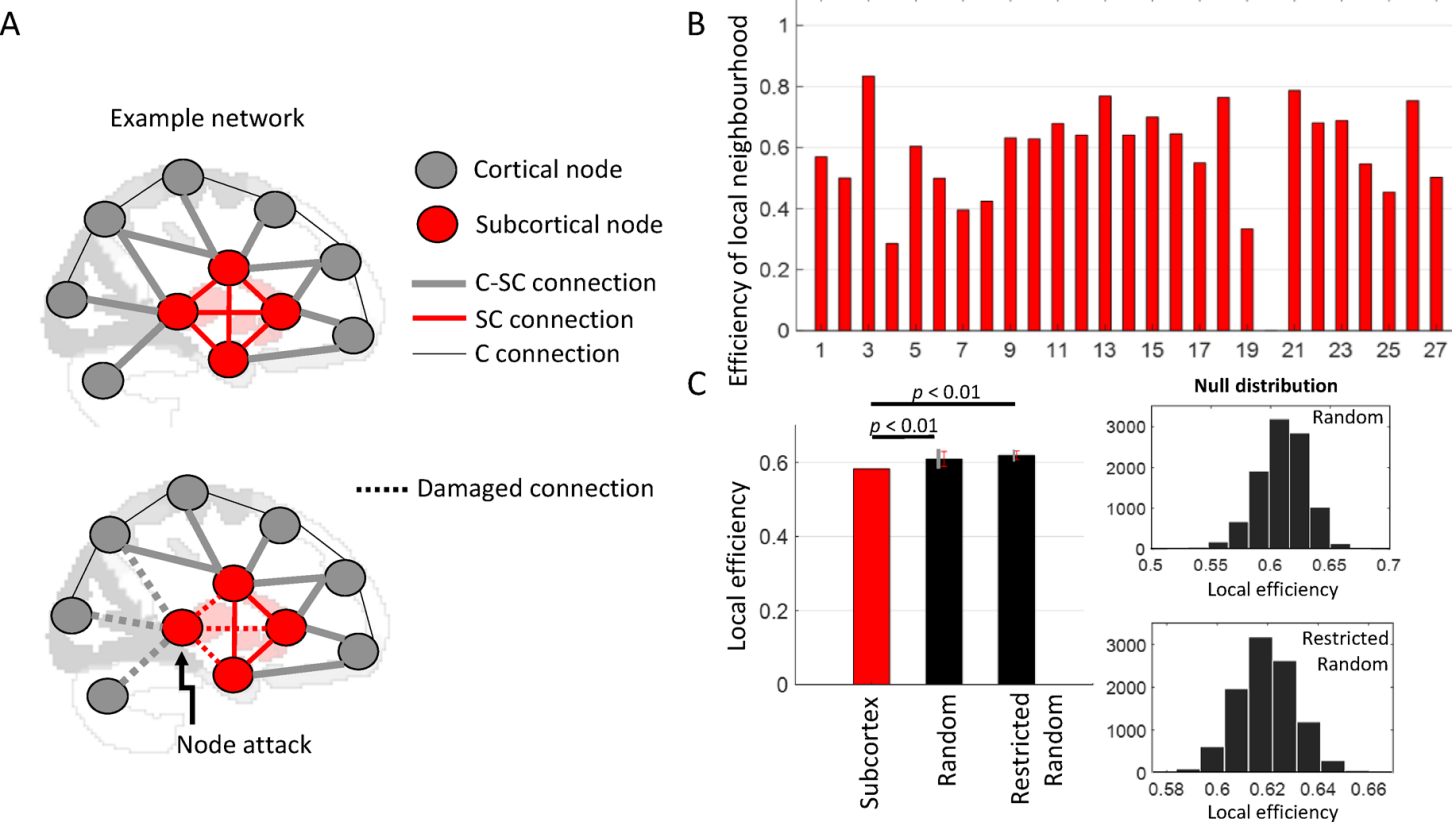
Subcortical nodes under attack. **(A)** shows an example network with cortical and subcortical nodes as well as different types of connections between – two cortical nodes (C connection), two subcortical nodes (SC connection), and a cortical node and a subcortical node (C-SC) connection. Also shown for illustration is attack on a subcortical node, where all its existing connections are removed (dashed line). (**B)** Efficiency of neighbourhood of a subcortical node, representing how efficiently the communication occurs between the immediate neighbours of the node, when that node is removed. (**C)** Local efficiency (LE) in case of targeted and random attack conditions, illustrating greater impact of subcortical damage on the efficiency of cortico-subcortical converging organization in comparison to random attacks (*p* < 0.01). Also shown are null distributions of LE values from 10,000 simulations in case of two random attack conditions. Error bars (in grey) indicate standard deviation of LE values across 10,000 random attack simulations.

We validated the indispensable role of a node in the cortico-subcortical converging organization by assessing the damage caused by an attack on that node, simulated here by removing the node (i.e., all its connections) (Fig. 7A). We quantified the potential impact of the attack by measuring its effects on the local information transfer efficiency of the attacked node. The local efficiency (LE) is average efficiency of local neighbourhoods and it shows how efficiently the immediate neighbours of a node communicate when the node is removed^49^ (see Methods, Node attack and local efficiency for more details). We simulated a targeted attack on 27 subcortical nodes and compared it with (10,000 simulations) on the converging organization. The neighbourhood of a subcortical node comprises of other subcortical nodes and “converging” cortical nodes. Figure 7B shows the efficiency of individual neighbourhoods due to attack on respective subcortical node. Additionally, we also simulated a more restricted random attack, in which we randomly selected a set of 27 cortical nodes of the converging organization. The latter condition was used to position the role of cortical nodes participating in the converging organization. We found that the local efficiency in case of targeted attack was lesser in comparison to that in any random attack condition (Fig. 7C; $\:{LE}_{target}$ = 0.57; mean $\:{LE}_{random}$ (± SD) = 0.61 (± 0.02), *t*[9999] = 167.35, *p* < 0.01; mean $\:{LE}_{restrand}$ (± SD) = 0.62 (± 0.01), *t*[9999] = 395.54, *p* < 0.01). This indicated a greater and significant impact of subcortex damage than any random damage to the converging organization.

random attacks.

## Discussion

The resting state organization within the cortex is configured to maintain a dynamic balance between the two fundamental organizational principles of local segregation and global integration^15,16,30^. While large-scale resting state organization of cerebral cortex and cerebellum has been studied well, the subcortical dynamics at rest remains poorly understood with respect to the fundamental organizational principles. Recent advances in neuroimaging data and availability of such data in public-domain^50^ have ushered the examination of the subcortical dynamics. In this study, using publicly-available multi-session resting state fMRI data from 92 healthy adults^50^ we identified the segregated networks within subcortex and further, revealed how these subcortical networks participate in global information integration via both subcortico-subcortical and cortico-subcortical communication. Elucidating the underlying subcortical dynamics at rest is crucial because it has been shown that large-scale cortical dynamics supports different cognitive abilities by transfer of task-related information via resting state connections^14,22^.

**Resting state networks of subcortex.** Using partial correlation based functional connectivity and bottom-up hierarchical clustering algorithm (Louvain), we reliably detected three segregated communities within subcortex – thalamic network, basal ganglia network, and subcortical limbic network (Figs. 3 and 4, Supplementary Figs. S1-S5). The subcortical networks reflected not only anatomical boundaries but also putative functional boundaries, which is in line with past studies suggesting that the strong functional connectivity between the nodes of a network is due to direct neuroanatomical connections existing between them^39,40^. We believe that these modular networks were driven by their well-known specialized functional roles. For instance, besides being primarily involved in multisensory as well as sensorimotor integration^19,51^ thalamic nuclei also involve in cognitive control, working memory, and attention^4^. In contrast, the basal ganglia is known to be primarily involved in motor control (deciding which actions to allow and which to inhibit) and additionally in reinforcement learning and executive decision-making in-concert with diverse frontal areas^3,7^. Distinct from both thalamus and basal ganglia, amygdala is primarily involved in processing emotionally salient stimuli and in coupling with hippocampus where emotional aspects along with sensory and cognitive dimensions of an experience are bound together for a unified memory encoding^5,6^.

**Subcortical hubs.** Subcortex exhibited equivalent topological characteristics as cortex and contained roughly four times more connectors than non-connectors (Fig. 4, Supplementary Table S1, S2). We observed that the connectors were positioned radially outward in the periphery of subcortex, including nodes in hippocampus, amygdala, putamen, dorsal caudate nucleus, nucleus accumbens, and dorsal, ventrolateral, posterior thalamus whereas the non-connectors were located inwards in the core of subcortex, including nodes in ventromedial thalamus, ventral caudate nucleus, and globus pallidus. This organization is intuitive given that the connectors participate in multiple communities across whole brain, whereas non-connectors including provincial hubs connect within their community only. Previous work has shown hippocampus, amygdala, putamen, caudate head, and thalamus as connector hubs^4,30,45^. Overall, this underscores that despite its deep and compact structure, subcortex contributes significantly in global brain communication and in shaping the large-scale dynamics^21^.

**Cortico-subcortical convergence at rest.** The main finding of this study is the existence of cortical convergence within subcortex at rest, revealed at both the region-level and network-level (Fig. 5; Table 1, Supplementary Table S3). The resting state cortico-subcortical converging organization in humans is along the lines of the classic parallel organization of cortico-subcortical circuits^7^ identified using anatomical tract-tracing data and cellular physiological recordings in non-human primates. The thalamic network had major convergence from primary networks (VIS, SOM, and CER). Interestingly, the maximum connections to thalamus were received from cerebellum, which is crucial for locomotion and balance^52^ as well as generation of appropriately timed motor response to sensory stimuli^53^. The remaining convergence was from association networks (DAN, VAN, CON, and DMN), indicating role of thalamic nuclei in cognitive control, working memory, and attention^4^ besides primary involvement in multisensory as well as sensorimotor integration^19,51^.

Further, the motor regions (of SOM, CER) and the association regions, including sensory association regions (of DAN, VAN, CON, DMN) converged onto the basal ganglia nuclei, indicating towards resting state interaction between regions involved in instrumental learning of new actions or sequences based on the environment, reinforcement history, and outcomes^20,54^. Strikingly, no direct connections were received from the early sensory and limbic cortices (VIS, LIM). However, we believe that any sensory input required for control and refinement of actions^55^ enters these circuits via two pathways – subcortico-subcortical connectivity between thalamus (THA-DA) and BGN nodes and converging connections from sensory association areas in cortex. Similarly, through connectivity with subcortical limbic structures, the BGN nodes provide a gateway for limbic drives to gain access and bias the cortico-basal ganglia circuits^11,56^. Together, this points toward the presence of cortico-basal ganglia circuits, which control and develop non-innate actions/action-sequences during goal-directed and habitual behaviours^11,54^.

Finally, association regions (of LIM, DMN, and VIS) majorly converged onto the subcortical limbic network, indicating resting state communication between regions involved in emotion, memory, and related behaviours^5,57^. Specifically, hippocampus received connections from perirhinal, entorhinal and parahippocampal cortices^58^ known to play a central role in memory encoding, where the perceptual, emotional, and cognitive dimensions of an experience are bound together within a spatiotemporal framework^5^. Moreover, the cortico-subcortical limbic circuits found in our study are along the lines of illustrious ‘limbic model’^6^ consisting of – the parahippocampal-hippocampal circuit associated with spatiotemporal dynamics and memory^5,58^ the default circuit involved in autobiographical memories and introspective thoughts consisting of episodic and semantic memory^59^and the temporo-amygdala-orbitofrontal circuit involved in integration of sensory signals including visceral sensations and emotion with semantic memory^60^.

**Functional diversity and node attack.** Interestingly, we found that the cortical regions from functionally diverse communities participated in the converging organization at rest (Fig. 6). For instance, this is in line with previous studies^17,61^ showing anatomical fibres from functionally distinct cortical regions converge within the striatum. These zones of convergence may serve as subcortical counterparts to cortical hubs, supporting not only integration but also distribution of information from various cortical sites that are involved in specialized computations of a single goal-directed process. For instance, different areas of the prefrontal cortex are involved in specialized subfunctions of a value based decision making process, i.e. valuation, action selection, feedback evaluation, and learning^62,63^. However, all these cortical areas must work in concert to create an optimal action plan to achieve the goal, likely with the help of the striatal areas. Further, the crucial role of subcortex for the converging organization was validated by decreased local efficiency within the organization during targeted attack condition in comparison to random attacks (Fig. 7). Thus, the subcortex serves as an appropriate anatomical substrate due to proximity of its many smaller nuclei^1^ supporting communication at higher information transmission efficiency between distinct functional systems sending progressively compressing pathways to subcortical nuclei^20^.

The finding of cortico-subcortical converging organization within the brain connectivity domain may offer valuable insights into how various brain disorders disrupt neural topology and function. Research has identified connectivity abnormalities across a broad spectrum of neurological and psychiatric conditions such as schizophrenia^64,65^ major depressive disorder^66^ dementia^67^, Alzheimer’s disease^68^, Parkinson’s disease^69^ and other brain disorders, see reviews^35,70^; each influencing the brain’s communication efficiency and capacity in distinct ways. Within the framework of our study, it is plausible to hypothesize that disorders impacting the subcortical converging zones may exert more widespread effects on neural communication, thereby impairing multiple cognitive functions.

There are some limitations of the work. First, the use of 3 T MRI data relatively limits the spatial and temporal resolution compared to higher-field MRI systems, such as 7T^71^. This constraint can hinder the detection of fine-grained functional networks and subtle connectivity patterns, particularly in subcortical or small cortical regions. Additionally, 3 T MRI is more susceptible to physiological noise and signal dropouts, especially in areas near air-tissue interfaces, which may reduce the reliability of connectivity estimates. These factors can limit the accuracy and sensitivity of connectivity analyses. Looking ahead, the use of ultra-high-field MRI (e.g., 7 T) offers a promising avenue to overcome these challenges by providing higher signal-to-noise ratios, improved spatial resolution, and better sensitivity to subtle neural activity. Second, resting state analyses do not provide direct empirical evidence towards how converging organization would support multiple cognitive functions. Current study has limitations by relying on resting state fMRI data alone without linking to task fMRI data. However, some of the past studies have shown transfer of diverse, task-related information through “resting state connections”^14,22^. Other empirical methods exist that can perform simulated lesioning with a direct link to task data, e.g. the activity flow network modelling approach that simulates task activations and behaviour from activity flowing over rest FC connections^72,73^. Future work can involve seeking convergence between the current rest-only approach and the rest-task activity flow approaches. This would make a more compelling case for the criticality of subcortical networks to neural communication.

In this study, we unveiled the subcortical configuration with respect to the local segregation and global integration principles of brain organization in humans. We identified the segregated resting state networks of subcortex and revealed their participation in cortical information integration at global scale. Future work will aim towards determining the re-configuration of the cortico-subcortical converging organization during task and diseased conditions.

## Methods

### Resting state fMRI data

In this study, we examined a publicly available resting state functional neuroimaging dataset from the Human Connectome Project (HCP)^50,74^. We used data from 92 participants (46 males and 46 females, age range = 22–35 years, mean (± SD) age = 29.49 (± 3.56) years) from the HCP 100 unrelated subjects’ release. All participants provided an informed consent, and the study protocols were approved by the Institutional Review Board at Washington University. All methods were performed in accordance with the relevant guidelines and regulations. Data were collected using a Siemens 3 T Connectom Skyra with a 32-channel head coil (detailed acquisition protocols are available here^74^. Participants were instructed to keep their eyes open and focus on a cross presented on a dark background. Resting state fMRI (rs-fMRI) data were acquired from four scan sessions of approximately 15 min each, over a period of two days, using a gradient-echo planar imaging sequence (parameters of imaging: TR = 720 ms, TE = 33.1 ms, flip angle = 52°, number of slices = 72, slice thickness = 2 mm, 2 mm isotropic voxel resolution, and multiband factor = 8). During each day, oblique axial acquisitions alternated between two phase encoding directions: right-to-left (RL) in one scan session and left-to-right (LR) in the subsequent scan session. We used data from scan sessions with LR phase encoding directions only.

### Preprocessing

We employed the minimally pre-processed, cleaned data (ICA-FIX clean dataset^75,76^. The minimal preprocessing pipeline (MPP) processed fMRI data by removing spatial distortions, realigning scans for subject motion, registering functional scans to the structural data, reducing the bias field, and normalizing the data to a global mean. Further, to maximize spatial correspondence between participants, the timeseries were mapped from a volume space to a standard “gray ordinate” space (more details can be found here^75^; followed by smoothing to regularize the mapping process. The primary goal of MPP was to retain as much relevant information as possible. However, further preprocessing techniques—such as spatial smoothing, temporal filtering, nuisance regression, and scrubbing—can inadvertently remove valuable neural signal activity as highlighted in previous studies^75^. Additionally, there remains ongoing debate regarding the best strategies for these techniques^77,78^ emphasizing the need for careful consideration based on specific research goals and data characteristics. Thus, we refrained from applying any more cleaning procedures than just implemented in ICA-FIX cleaning procedure. The ICA-FIX process removes structured spatial/temporal artefacts and motion fluctuations from the data^76^. All further analyses were carried out using custom-written routines in MATLAB software (Version 9.11.0.1809720 (R2021b) Update 1), including functions from Brain Connectivity Toolbox^79^.

### Whole brain functional parcels

We used a combination of functional parcellation schemes defining 400 cerebral cortex parcels (200 per hemisphere) from Schaefer atlas^80^ 26 cerebellar cortex regions from Automated Anatomical Labelling (AAL) atlas (9 bilateral divisions and 8 medial divisions (Vermis))^81^ and 54 subcortex parcels (27 per hemisphere) from a recent multiscale subcortical atlas^25^. We refer cerebral cortex and cerebellar cortex commonly as cortex, unless otherwise mentioned specifically. Supplementary Fig. S1 shows 400 cerebral cortex parcels as part of 7 canonical resting state networks according to^23^ including visual network (VIS), somatomotor network (SOM), dorsal attention network (DAN), ventral attention network (VAN), limbic network (LIM), control network (CON), and default mode network (DMN). Additionally, the figure shows cerebellum (CER) consisting of 26 parcels and 7 subcortical regions (thalamus, putamen, caudate nucleus, nucleus accumbens, globus pallidus, hippocampus, and amygdala) consisting of 54 parcels.

### Regions-of-interest

Resting state network organization in human brain is highly symmetrical^23,24,82^. Thus, for further analyses we considered the symmetrical bilateral parcels for an individual brain area as one region-of-interest (ROI). This resulted into a total of ($\:N$ =) 244 ROIs, which included 200 cortical ROIs, 17 cerebellum ROIs, and 27 subcortical ROIs. For each participant, we extracted the summary (mean) time courses for each ROI from the ICA-FIX cleaned data. We discarded the first 100 volumes (approx. 72 s) from each of the two LR phase encoding scan sessions (completed over two days) because the first few volumes of a functional acquisition contain large signal changes which stabilise as the tissues reach steady state^83^. Further, the time courses from individual sessions were mean-centred to remove the mean intensity difference across sessions and then concatenated^29^.

### Functional connectivity

To reveal underlying network organization at rest and compute network-based metrics, a whole brain functional connectivity^84^ (FC) matrix was determined based on a common statistical modelling approach^37,85,86^. We quantified the *partial* pair-wise correlation between ROIs by using a multiple linear regression model. Partial correlation captures unique dependence between any two nodes while controlling for the effects of all other nodes in consideration (i.e. subcortical as well as cortical nodes). For each participant, we estimated the mean temporal activity from a ROI, say $\:{X}_{i}$, based on the activity of remaining $\:N-1$ (= 243) ROIs. This was done by including the remaining ‘$\:N-1$’ time courses as regressors in the model. In this way, activity in a region $\:{X}_{j}$ explained the activity or captured the partial variance in the estimated time course $\:{X}_{i}$ uniquely due to itself and the shared variance due to remaining regressors, say $\:{X}_{k}$, was not attributed to the regressor $\:{X}_{j}$, where, $\:i,j,k\in\:\left\{\text{1,2},\dots\:,N\right\}\:$and$\:\:i\ne\:j\ne\:k$. Thus, the parameter estimates $\:{\beta\:}_{j,i}$ (coefficient of regressor $\:{X}_{j}$ in the model estimating $\:{X}_{i}$) provided a weighted measure of unique or partial influence of the activity in ROI $\:{X}_{j}$ on the activity in ROI $\:{X}_{i}$, by controlling for the influence from other ROIs $\:{X}_{k}$. The higher the magnitude of the parameter estimates, the greater the correlation between the regions. Using the above approach helped controlling for any false positives. Self-dependence was not modelled based on the method used in^37,85^. No intercept term was included in the regression model as the regional time courses were mean-centred. For each participant, we repeated the above estimation procedure for each of the $\:N$ whole brain ROIs.

*For group-level significance*, individual parameter estimates from each participant were subjected to one-sample *t*-test (a random effects analysis method^87^. For all reported results, we employed a significance threshold of *p <* 0.05, Bonferroni corrected for multiple comparisons (equal to number of regressors in the model as the activities of the nodes are not fully independent^43,88^. Finally, we computed a group-level whole brain *binary* functional connectivity matrix $\:C\:$(of size $\:N\times\:N$), where the element $\:{C}_{j,i}$ is 1, if activity in ROI$\:\:{X}_{j}$ had significant influence on the activity in ROI $\:{X}_{i}$; otherwise, 0. The connectivity matrix was asymmetrical as $\:{C}_{j,i}$ may or may not equal $\:{C}_{i,j}$. We also computed a group-level whole brain *weighted* functional connectivity matrix $\:wC$, where the elements of the matrix were significant parameter estimates from the models, i.e., $\:{wC}_{j,i}$ is $\:{\beta\:}_{j,i}$, if activity in ROI$\:\:{X}_{j}$ had significant influence on the activity in ROI $\:{X}_{i}$; otherwise, 0. Thereafter, we normalized the weights in the matrix, such that they ranged between $\:-1\:to\:1$.

### Subcortical community detection

#### Louvain clustering algorithm

To determine whether and how parcels in subcortex formed functionally segregated communities at rest, we employed the Louvain clustering algorithm^38^. A community consists of a subset of unique nodes, which have stronger connections within the community in comparison to outside than expected in a random “null” network, resulting into nonoverlapping partitions or modules of a network, for more details refer^89^. The degree of modularity in a network can be characterized by the *Q* index^90,91^. The Louvain algorithm operates as follows: it begins by identifying smaller communities through optimization of a modularity function *Q*, which is primarily based on the connectivity matrix of the network nodes, a resolution parameter $\:\gamma\:$, and an initial random community assignment. Next, it merges these smaller communities into larger ones, creating a new network. This iterative process continues until there are only minimal changes in the value of modularity index *Q*. The algorithm aims on enhancing the strength of connections within a community rather than the connections between the communities^38^.

We determined the functional networks in subcortex based on the group-level binary functional connectivity matrix of 27 subcortical ROIs (of size 27 × 27) computed in previous section. The resolution parameter $\:\gamma\:$ was set to standard value of 1 and 1000 random initializations of community assignment were made. The resolution parameter $\:\gamma\:$ controls the number of community partitions, where $\:\gamma\:<1$ partitions a network into fewer (but larger) nonoverlapping communities and $\:\gamma\:>1$ into more (but smaller) nonoverlapping communities than standard ($\:\gamma\:=1$). The resolution parameter $\:\gamma\:$ was varied around the standard value of 1, i.e. from 0.0 to 2.0 in steps of 0.1.

For individual $\:\gamma\:$ value, 1000 random initializations of community assignment were made. Even for a single $\:\gamma\:$ value there are multiple possible solutions that can optimize the modularity function *Q*, resulting into partitioning outcomes/solutions that vary across different random initializations of community assignment. To control for this variability, we performed clustering 1000 times each starting with a random community assignment and thereafter, employed a consensus approach across all 1000 partitioning outcomes^89,92^. The consensus approach computed one final partitioning scheme using the Louvain algorithm on an agreement matrix (27 × 27; like a connectivity matrix) and a resolution parameter $\:\tau\:$. The agreement matrix contained the probability of each pair of nodes to be assigned to the same community across all 1000 outcomes. The consensus approach used a parameter $\:\tau\:$ which served as a threshold value for the agreement matrix to control the resolution of final partitioning scheme. We set the value of $\:\tau\:$ to 0.95, so that the pair of nodes which have been consistently partitioned into the same community more than 95% of the times across all 1000 outcomes were included into the computation of final partitioning scheme for a given $\:\gamma\:$ value.

We determined the best $\:\gamma\:$ value and thus, the best partitioning solution by assessing the network assignment confidence scores at all $\:\gamma\:$ values. We computed the network assignment confidence score for a given $\:\gamma\:$ value as mean of the Silhouette scores of subcortical nodes, explained next. The higher the network assignment confidence score, the better the final partition solution. We refer to the best final partition solution as the “representative” resting state network organization in subcortex.

### Silhouette score

The silhouette score $\:{S}_{i}\:$for a node $\:i$ evaluates its closeness (a measure of distance in terms of connectivity strength - correlation) to the other nodes in the same community compared to the nodes in the next nearest community^93^. The higher the connectivity strength between two nodes, the closer the two nodes. The score was computed using weighted partial connectivity matrix *wC* of subcortical nodes (27 × 27), as shown below:

1 $$\:{S}_{i}=\:\frac{{b}_{i}-{a}_{i}}{\text{m}\text{a}\text{x}({a}_{i},{b}_{i})}$$

where, $\:{a}_{i}$ is the average distance from node $\:i$ to the other nodes in the same community as node $\:i$, and $\:{b}_{i}$ is the minimum average distance from node $\:i$ to the nodes in a different community, minimized over subcortical communities. The silhouette score for each node ranges from − 1 to 1, where a positive value signifies greater confidence in the current community assignment. Conversely, a negative value indicates stronger association on average to the next nearest community than its assigned community. If most nodes have a high silhouette score, then the clustering solution is appropriate. If many nodes have a low or negative score, then the clustering solution indicates too many or too few communities. Next, we performed a more rigorous validation of our results.

### Statistical network validation

Statistics-based network analysis^43,44^ was used to test whether the obtained clustering solution, i.e. the three segregated communities in subcortex did not occur by chance. Characteristic graph theory metrics were computed for the real and random networks both at node-level and network-level, including measures of segregation (within-module degree (WMD), modularity index $\:Q$) and those of integration (participation coefficient (PC), characteristic path length (CPL)). For computation of all graph metrics used here, we employed the group-level binary partial connectivity matrix (27 × 27). We briefly describe each metric next (for implementations and more details refer^34,79^. The measures of segregation characterize the community structure and communication within a community, where within-module degree $\:{wmd}_{i}$ is the number of connections $\:{k}_{im}\:$of node $\:i\:$in its own community $\:m$. A corresponding z-score metric $\:{z}_{i}\:$shows how well-connected is node $\:i\:$within its own community $\:m$ in comparison to other nodes of its own community, as shown below:

2 $$\:{z}_{i}=\frac{{k}_{im}-\stackrel{-}{{k}_{m}}}{{\sigma\:}_{m}}$$

where, $\:{k}_{im}\:$denotes the number of within-module connections (bidirectional – incoming and outgoing) of a node$\:\:i\:$in its community $\:m$, $\:\stackrel{-}{{k}_{m}}$ and $\:{\sigma\:}_{m}\:$are the average and standard deviation values across the number of within-module connections of all nodes in community $\:m$, respectively. The modularity index $\:Q$, a network-level segregation metric, indicates on average how well-connected are the communities within themselves in comparison to each other^89^. A $\:Q$ value ranges from 0 to 1, where a value close to 0 suggests that the network resembles a random network, while a value close to 1 indicates a strong community structure.

The measures of integration characterize communication between the communities, where participation coefficient $\:{P}_{i}\:$indicates how well-connected is a node to all communities of the network, as shown below:

3 $$\:{P}_{i}=1-\sum\:_{m=1}^{{N}_{m}}{\left(\frac{{k}_{im}}{{k}_{i}}\right)}^{2}$$

where, $\:{N}_{m}$ is the number of communities, please note, in the subcortex only, $\:{k}_{i}\:$is the degree of node $\:i$ (total number of bidirectional connections – incoming and outgoing) and $\:{k}_{im}$ is the number of connections between node $\:i$ and nodes of any community $\:m$. If a node’s connections are entirely restricted to its own community, its PC is 0; while a node with high PC (closer to 1) connects to multiple communities. The characteristic path length $\:\lambda\:$, a network-level integration metric, is the average shortest distance between all pairs of nodes in a connected network. A lower CPL value indicates higher integration between communities resembling a random network.

For each of the four characteristic graph metrics (i.e., within-module degree, modularity index, participation coefficient, and characteristic path length), we obtained a “null” distribution of metric coefficients from a population of 1000 random networks. Please note that in case of a node-level metric, say WMD, 27 null distributions were computed each corresponding to the 27 nodes in the subcortex. Then, using one-sample *t*-tests we checked whether the real network’s metric values were significantly different from their respective null distributions. For all reported results, we employed a significance threshold of *p <* 0.01, Bonferroni corrected for multiple comparisons as the activities of the nodes are not fully independent^43,88^.

### Topology of subcortex

To understand the role of subcortex in local segregation and global integration dynamics, we examined its topology using network science metrics^34^. Specifically, we identified four distinct types of nodes in subcortex, including connector hubs, non-hub connectors, provincial hubs and non-hubs. Connectors hubs are brain regions which have many connections both within their community and outside with multiple communities and non-hub connectors are regions with more connections in other communities in comparison to connections within their community, whereas provincial hubs are regions which have many connections within their own community^15,45,46^. We classified the 27 subcortical nodes based on their participation coefficient and WMD z-score (see Eqs. (2), (3) in previous section). We used a total of ($\:{N}_{m}$ =) 11 whole brain community partitions (covering *N =* 244 ROIs), including the 3 subcortical and 8 cortical communities, and group-level binary FC matrix (of size 244 × 244). A node $\:i$ was classified as “connector hub” if its participation coefficient $\:{P}_{i}>0.5\:$and WMD z-score $\:{z}_{i}>0$; as “non-hub connector” if $\:{P}_{i}>0.5\:$and $\:{z}_{i}\le\:0$; as “provincial hub” if $\:{P}_{i}\le\:0.5\:$and $\:{z}_{i}>0$; and as “non-hub” if $\:{P}_{i}\le\:0.5\:$and $\:{z}_{i}\le\:0$ (using Eqs. (2), (3) with $\:{N}_{m}$ = 11)^34,47^. The percentage of a particular node type (say, connectors in subcortex) was calculated by dividing the number of that node type with the total number of nodes (in the subcortex).

Further, we compared the topology of subcortex with that of cortex based on the following characteristic graph metrics^34^ – degree (absolute number of connections), within-module degree, connection density (degree per voxel), clustering coefficient (of a node is local graph metric indicates how well the neighbours of that node are connected), and participation coefficient (a global graph metric). In addition to comparing the absolute number of connections (degree) across node types in subcortex and cortex, we also compared their connection densities to test whether subcortex can support functional integration equivalent to cortex, despite its smaller nuclei structures. The connection density of a node was measured by dividing its degree with the size of the region, i.e. the number of isotropic voxels (2 mm x 2 mm x 2 mm) contained in the region. Some of the metrics are described in previous section.

### Cortico-subcortical converging organization

#### Convergence and distance metrics

To determine the extent of convergence in the subcortex, we computed the absolute number $\:{N}_{s}^{conv}\:$of cortical ROIs converging (via bidirectional connections) onto a subcortical ROI within the subcortex as below:

4 $$\:{N}_{s}^{conv}=\sum\:_{r\ne\:s}{c}_{r,s}$$

Where, $\:{c}_{r,s}=\:1,\:$if a bidirectional (symmetric) connectivity exists between cortical node $\:r$ and subcortical node $\:s$ and $\:{c}_{r,s}=0$ otherwise, computed using the group-level whole brain binary graph $\:C=\left\{{C}_{r,s}\right\}$ (of size 244 × 244) at a significance threshold of *p <* 0.05, Bonferroni corrected for multiple comparisons. We considered bi-directional connectivity between nodes because “dual dyad” motif (a subgraph of three nodes having two sets of bidirectional connections joining at a third single node) have been found to be present in abundance in larger network as compared to other possible subgraphs^94^. Motifs are building blocks of a larger network and unfold underlying statistical properties of structural and functional brain organization^94,95^. Also, it seems biologically plausible to have a dual dyad motif in abundance, given that not only integration but also distribution of information is crucial for efficient brain function.

To determine how convergence between the cortical nodes was related to the distance between the nodes and the types of nodes (functionally similar or diverse) in cortex, we computed proportion convergence $\:{O}_{i,j}\:$between cortical nodes $\:i$ and $\:j$. In past, a similar analysis just based on distance has been done for tract-tracing data from non-human primates^18^ but is still missing for non-invasive human resting state data. We categorized a cortical node pair as functionally similar, if both its nodes belong to a same RSN and as functionally diverse, if its nodes belong to distinct RSNs. The metric $\:{O}_{i,j}\:$is the proportion of convergence for the cortical node pair $\:(i,j)$ with respect to the total convergence, calculated as below^18^:

5 $$\:{O}_{i,j}=\frac{\sum\:_{s\ne\:i,j}{\delta\:(c}_{i,s},{c}_{j,s})}{{\sum\:}_{i,j}{O}_{i,j}}$$

Where, $\:\delta\:(x,y)$ is the Kronecker delta that is 1 if x is equal to y and 0 otherwise, $\:s\:$denotes a subcortical node, $\:{c}_{r,s}=1$, if a bidirectional connectivity exists between cortical node $\:r$ and subcortical node $\:s$, and 0 otherwise, as aforementioned. The metric value lies between 0 and 1 and captures the frequency of convergence, i.e., a higher metric value indicates that a cortical node pair converges at multiple subcortical nodes.

Next, we computed the Euclidean distance (in mm) between the cortical parcels $\:i$ and $\:j$ as below:

6 $$\:{D}_{i,j}=\sqrt{{({x}_{ci}-{x}_{cj})}^{2}+{({y}_{ci}-{y}_{cj})}^{2}+{({z}_{ci}-{z}_{cj})}^{2}}$$

Where, $\:({x}_{cr},{y}_{cr},{z}_{cr})$ denotes the coordinates of the centroid of a brain parcel $\:r$. Since our ROIs (nodes) are formed using bilateral parcels, to compute distance between two cortical ROIs we averaged over the distance between respective parcels in left hemisphere and the distance in right hemisphere. To obtain distribution of distances, we divided the cortical node pairs into bins of equal distance range, i.e. 8 mm bin sizes, and summed the proportion convergence of the cortical node pairs falling into a particular bin.

#### Node attack and local efficiency

To quantify the potential effects of attack on a set of nodes, we measured the local efficiency of the attacked nodes^49^. The local efficiency (LE) is average efficiency of $\:{N}_{G}$ local subgraphs or neighbourhoods. The efficiency $\:E\left({G}_{i}\right)\:$of a local neighbourhood $\:{\:G}_{i}\:$is the strength of communication among the immediate neighbours of node$\:\:i$, when node $\:i$ is removed, computed as below:

7 $$\:E\left({G}_{i}\right)=\frac{1}{{N}_{i}({N}_{i}-1)}{\sum\:}_{j\ne\:k\in\:{G}_{i}}\frac{1}{{l}_{jk}}$$

Where,$\:\:i=1,\dots\:,\:{N}_{G}$, $\:{G}_{i}$ is the neighbourhood of node $\:i$, $\:{N}_{i}\:$is the number of nodes in neighbourhood$\:\:{G}_{i}$, $\:{l}_{jk}\:$is the shortest path length between nodes $\:j,k\:(\ne\:i)$ of the neighbourhood $\:{G}_{i}$. A path in a network graph is defined as the minimum number of distinct edges that must be traversed to pass from one node to another in the network^34,43^. It is a measure of how efficiently two nodes can transmit information to each other. A lesser LE value indicates, on average, poor communication in the local neighbourhoods and thus, a greater impact of attack.

We simulated two types of attacks^96^: (1) a targeted attack which refers to damage inflicted on a particular set of nodes/connections in the network, and (2) a random attack which refers to damage inflicted on a set of randomly selected nodes/connections in the network. Additionally, we also simulated a more restricted random attack, in which we randomly selected a set of 27 cortical nodes of the converging organization. Further to compare the effects of targeted attack, we generated a null distribution of local efficiency values by simulating 10,000 random attacks. Specifically, we used a one-sample t-test with null hypothesis that data of 10,000 LE values comes from a distribution with mean equal to the local efficiency of target attack. A rejection of null hypothesis at *p* < 0.01 would indicate that there is significant difference in the effects of targeted attack and that of random attack.

## Supplementary Information

Below is the link to the electronic supplementary material.

Supplementary Material 1

## Data Availability

The dataset used in the current study is publicly available from Human Connectome Project. The source data and source code (.m) files used for analysis and generation of results in the current study will be uploaded on Figshare repository and subsequently, will be made public after publication of the study.
